# Quality of life of women with recurrent vulvar cancer treated with electrochemotherapy

**DOI:** 10.2478/raon-2025-0019

**Published:** 2025-03-19

**Authors:** Gregor Vivod, Ines Cilensek, Nina Kovacevic, Gregor Sersa, Maja Cemazar, Sebastjan Merlo

**Affiliations:** 1 Department of Gynecological Oncology, Institute of Oncology Ljubljana, Ljubljana, Slovenia; 2 Faculty of Medicine, University of Ljubljana, Ljubljana, Slovenia; 3 Institute of Histology and Embryology, Faculty of Medicine, University of Ljubljana, Ljubljana, Slovenia; 4 Faculty of Health Care Angela Boskin, Jesenice, Slovenia; 5 Department of Experimental Oncology, Institute of Oncology Ljubljana, Ljubljana, Slovenia; 6 Faculty of Health Sciences, University of Ljubljana, Ljubljana, Slovenia; 7 Faculty of Health Sciences, University of Primorska, Izola, Slovenia; 8 Faculty of Medicine, University of Maribor, Maribor, Slovenia

**Keywords:** electrochemotherapy, vulvar cancer, recurrence, bleomycin, quality of life

## Abstract

**Background:**

The quality of life of patients undergoing oncologic treatment has become an important issue in recent years. Owing to potential mutilation following surgery for vulvar cancer, more conservative approaches have evolved with the integration of new local ablative therapies, such as electrochemotherapy. The aim of this study was to determine the quality of life of women with vulvar cancer recurrence treated with electrochemotherapy for nonpalliative purposes.

**Patients and methods:**

Eleven patients with vulvar cancer recurrence were treated with electrochemotherapy from July 2020 to December 2023. Patients completed different questionnaires: the EuroQol - 5 Dimension (EQ-5D), European Organization for Research and Treatment of Cancer Quality of Life Questionnaire Core 30 (EORTC QLQ-C30), European Organization for Research and Treatment of Cancer Quality of Life Questionnaire Vulva Cancer 34 (EORTC QLQ-VU34) and visual analog pain scale (VAS) before and one, three and six months after electrochemotherapy. As a control group, fifteen patients with vulvar cancer recurrence treated with wide local excision completed the EORTC QLQ-C30 and VAS questionnaires before surgery and three and six months after surgery.

**Results:**

No significant differences in EQ-5D scores were found between quality of life before electrochemotherapy and at each follow-up visit. A comparison of the EORTC QLQ-C30 scores between the electrochemotherapy and surgery groups showed a significant difference in physical functioning, fatigue, insomnia, and global health status three months after the procedure and in role, cognitive, social functioning and appetite loss six months after the procedure, all of which were in favor of the electrochemotherapy group. The EORTC QLQ-VU34 questionnaire showed improvements in urinary symptoms and symptoms related to scarring and mutilation of the external genitalia in the electrochemotherapy group. The VAS score did not differ significantly between the electrochemotherapy and surgical groups.

**Conclusions:**

The study showed that the quality of life after treatment with electrochemotherapy is better in some segments than after surgical treatment.

## Introduction

Vulvar cancer is a rare malignant disease that accounts for 4–5% of all gynecologic cancers and 0.3% of all cancers.^1,2^ Risk factors for vulvar cancer include increasing age, infection with human papillomavirus, inflammatory diseases of the vulva, immunodeficiency and cigarette smoking.^3^ In the last decade, a significant increase in the incidence of vulvar cancer has been observed in younger women worldwide, which can be attributed to infection with the human papillomavirus.^2,4,5^ More than 90% of cases of vulvar cancer are squamous cell carcinomas. Other pathological subtypes include vulvar melanoma (5–7%), vulvar basal cell carcinoma (2–4%), and vulvar extramammary Paget’s disease (1–3%).^2,6^

The decision on the treatment of vulvar cancer depends on the stage, the age of the patient, the performance status, and the presence of concomitant diseases.^7^ The main treatment modalities for vulvar cancer are surgery and radiotherapy.^8^ Local recurrences of vulvar cancer are reported in 40% of patients with early-stage disease in the first 10 years of follow-up, and a second recurrence is reported in approximately 20% of these patients.^9,10^ In patients with a first local recurrence of vulvar cancer, the disease-specific survival rate decreases by 20% and even further decreases after additional recurrences.^10,11^ The treatment of local recurrences is usually challenging. The aim of surgery is to achieve a free tumor surgical margin status, which often leads to radical procedures with partial resection of the urethra, anus, clitoris and vagina. Radical surgery in the vulvar area can therefore lead to scarring and mutilation of the external genitalia.^12,13^ Owing to the potential for mutilation and morbidity following surgery for local recurrence of vulvar cancer, the approach to treating vulvar cancer has evolved from invasive surgery to more conservative approaches that are as individualized as possible and incorporate new surgical techniques.^14,15^ In addition to the new surgical techniques, non-thermal ablative therapies, i.e. electrochemotherapy, have also proven effective in the treatment of such recurrences.^16^

In electrochemotherapy, short electric high-voltage pulses are used to improve the local administration of chemotherapeutic agents.^17^ The procedure, which is easy to use and relatively inexpensive compared with other alternatives, involves an intravenous bolus infusion of bleomycin or an intratumoral injection of bleomycin or cisplatin.^18,19^ Electrochemotherapy is already used for the treatment of superficial tumors such as melanoma, squamous cell carcinoma, basal cell carcinoma and sarcoma.^20–23^ It is also used to treat deep-seated tumors such as colorectal cancer, colorectal liver metastases and primary hepatocellular carcinoma.^24–28^ The unique advantage of electrochemotherapy over surgery is its selectivity for cancer cells while sparing surrounding healthy tissue.^29^ The use of electrochemotherapy in patients with vulvar cancer has been described in only a few studies or clinical cases and only for palliative purposes.^30–34^ Recently, our group used electrochemotherapy for the first time to treat the recurrence of vulvar cancer for nonpalliative purposes. The data from our studies have shown that electrochemotherapy is a feasible, safe and effective technique for the treatment of vulvar cancer recurrence.^13,16^

To the best of our knowledge, few studies address the impact of vulvar cancer treatment on patients’ quality of life.^12^ Vulvar cancer can cause severe pain and bleeding and lead to limitations in daily activities. After radical surgery in the vulvar area, women can develop urinary and fecal incontinence, which can cause severe discomfort and lead to a deterioration in quality of life and psychosocial well-being as well as social isolation.^35,36^ Potential problems related to sexuality are rarely addressed preoperatively, as disease prognosis is of paramount importance.^12,37^ In recent years, the quality of life of patients undergoing surgical or other oncological treatments for various types of cancer has become a central topic of cancer care.^14,38,39^

Data from previous studies have shown that electrochemotherapy can be used as a modern therapeutic tool for patients who would otherwise have to undergo surgery involving mutilation of the external genitalia.^13^ Electrochemotherapy also appears to be less mutilating and could be a promising alternative treatment for vulvar cancer recurrence, especially in cases where the clitoris, vagina, urethra and anus are in close proximity.^16^ The aim of the present study was to determine the quality of life of women with vulvar cancer recurrence treated with electrochemotherapy for nonpalliative purposes and to compare the quality of life of women treated with electrochemotherapy for nonpalliative purposes with the quality of life of women treated surgically.

## Patients and methods

### Study design

The study of the quality of life of women treated with electrochemotherapy for vulvar cancer recurrence for nonpalliative purposes was prospective and institution-based. The study was conducted at the Institute of Oncology Ljubljana. The study was approved by the Institutional Medical Board (number ERID–KSOPKR–0042/2021) and the Slovenian National Ethics Committee (number 0120–262/2021/3). The study has been registered at ClinicalTrials.gov under the identification number NCT05916690. All patients who participated in the study were presented to an interinstitutional tumor board consisting of a gynecologic oncologist, a radiation oncologist, a radiologist, a medical oncologist and a pathologist. A signed informed consent form was obtained from all patients who participated in the study.

### Patients and data collection

Eleven patients with vulvar cancer recurrence were treated with electrochemotherapy from July 2020 to December 2023. Patients were included in the study according to the inclusion and exclusion criteria.^13^ The data collected were anonymized to protect the privacy of all patients.

Patients treated with electrochemotherapy completed the following questionnaires to assess their quality of life before electrochemotherapy and one, three and six months after electrochemotherapy: EuroQol - 5 Dimension (EQ-5D);^40^ European Organization for Research and Treatment of Cancer Quality of Life Questionnaire Core 30 (EORTC QLQ-C30);^41^ European Organization for Research and Treatment of Cancer Quality of Life Questionnaire Vulva Cancer 34 (EORTC QLQ-VU34); and the visual analog pain scale (VAS).^42^

As a control group, 15 patients with vulvar cancer recurrence who were treated with wide local excision from January 2018 to January 2023 completed the following questionnaires to assess their quality of life before and three and six months after surgery: EORTC QLQ-C30^41^ and VAS.^42^

The EQ-5D is a standardized measure of health status developed by the EuroQol Group to provide a simple, generic measure of health for clinical and economic appraisal. The questionnaire covers five domains: mobility, self-care, usual activities, pain/discomfort and anxiety/depression. Each dimension has three levels (no problems, some problems, and major problems). The patients indicated their health state by ticking the box that marks the most appropriate level of problems in each dimension. A five-digit patient profile was obtained. The health states defined in this way were converted into a single summary index by applying a formula that assigns values to each of the levels in each dimension. A single summary index was calculated on the basis of the preference values of the population in Slovenia. The index ranges from -0.498 (lowest quality of life) to 1 (highest quality of life).^40,43^

The EORTC QLQ C30 was used as a cancerspecific questionnaire with 30 items to assess the general quality of life of patients with vulvar cancer. The EORTC QLQ-C30 includes five domains of functioning (physical, role, cognitive, emotional and social), three symptom scales (fatigue, pain and nausea/vomiting), single items that capture additional symptoms frequently reported by cancer patients (dyspnea, insomnia, loss of appetite, constipation, and diarrhea), the perceived financial impact of the illness and treatment and a general quality of life scale. All scales and individual values range from 0 to 100, with a high scale value indicating a high response level. For example, a high value for a functioning scale indicates a high/healthy level of functioning, a high value for global health/quality of life indicates a high quality of life, but a high value for a symptom scale/item indicates a high level of symptoms/problems.^41^

The EORTC QLQ-VU34 questionnaire was developed to measure the quality of life of patients with vulvar cancer. The instrument is currently undergoing phase four field validation by the EORTC group, but a preliminary version is already available. Permission was obtained for its use in the present study. For our study, we used preliminary items covering problems with urination (“Have you passed urine frequently?”, “Have you had pain or a burning feeling when passing urine?”, “Have you had leaking of urine?”, “When you felt the urge to pass urine, did you have to hurry to get to the toilet?”) and defecation (“Have you had leaking of stools?”, “When you felt the urge to move your bowels, did you have to hurry to get to the toilet?”), scarring and mutilation of the external genitalia (“Have you had narrowing/tightness of your vaginal entrance?”, “Has scarring in your genital area caused you problems?”, “Have you had difficulties sitting due to problems in your genital area?”), feelings about physical attractiveness (“Have you felt physically less attractive as a result of your disease or treatment?”, “Have you felt less feminine as a result of your disease or treatment?”, “Have you been dissatisfied with your body?”) and problems with sexual intercourse (“Have you been sexually active?”, “Have you worried that sex would be painful?”, “Have you had pain during sexual intercourse or other sexual activity?”, “Has your vagina felt narrow and/or tight during sexual intercourse or other sexual activity?”, “Has your vagina felt dry during sexual intercourse or other sexual activity?”, “Has sexual activity been enjoyable for you?”). The total score of the items was calculated by summing the scores of the individual items and dividing the sum by the number of items. Higher scores represent more symptoms and therefore a worse quality of life.^44^

The VAS is a valid and reliable measure of pain intensity that represents the severity of symptoms on a scale from 0 (“no symptoms”) to 10 (“very severe symptoms”).^42^

### Treatment procedures

All patients in both groups had a recurrence of a single local squamous cell vulvar carcinoma, which was confirmed by an experienced pathologist. Appropriate imaging was performed to exclude regional and distant metastases. Treatment with electrochemotherapy was performed according to standard operating procedures.^13,16,45^ Bleomycin was administered intravenously at a dose of 10,000 or 15,000 IU/m^2^, depending on the patient’s age and concomitant diseases.^46^ Eight minutes after the intravenous administration of bleomycin, electrical impulses were applied to the tumors via specially designed electrodes such that the entire tumor node was covered, including a safety margin of one centimeter. The electrical impulses were delivered via the Cliniporator VITAE (IGEA S.p.A., Carpi, Italy). In the control group, a wide excision of the tumor was made with a macroscopic safety margin of one centimeter.

### Statistical analysis

Statistical analysis was performed via IBM SPSS Statistics 27.0 software (Armonk, NY: IBM Corp.). Continuous variables were compared between groups via the independent samples t-test. The results for continuous variables are presented as the means with standard deviations (SD). Categorical variables were analyzed using the Chi-square test or Fisher’s exact test, depending on the expected frequency in each category. To analyze the EQ-5D, the EORTC QLQ-C30 and the EORTC QLQ-VU34 questionnaires, comparisons between subgroups were performed via the Mann–Whitney test (two groups) or the Kruskal–Wallis Z test (multiple comparisons). For the VAS score, the data were compared via the independent Student’s t-test. Statistical significance was set at p<0.05.

## Results

All 11 patients treated with electrochemotherapy completed the EQ-5D questionnaire, the EORTC QLQ-C30 questionnaire, the EORTC QLQ-VU34 questionnaire and the VAS questionnaire before electrochemotherapy and one, three and six months after electrochemotherapy. All 15 patients with vulvar cancer recurrence treated with wide local excision completed the EORTC QLQ-C30 questionnaire and the VAS questionnaire before surgery and three and six months after surgery.

The detailed data of the patients and the tumor characteristics are shown in Table 1 and were analyzed and described in our previous work.^16^ The electrochemotherapy group and the surgery group were homogeneous. There were no statistically significant differences between the electrochemotherapy group and the surgical group in terms of the mean body mass index, associated diseases, previous treatments, presence of lichen sclerosus or p16 status.

**TABLE 1.** Baseline characteristics of patients treated with electrochemotherapy and surgery

Characteristics	ECT (N = 11)	Surgery (N = 15)	p-value
Age at treatment (years; mean ± SD)	78.4 ± 9.0	67.8 ± 8.9	**0.006**
BMI (mean ± SD)	26.1 ± 3.9	26.8 ± 5.9	0.758
Duration of procedure (minutes, mean ± SD)	17.6 ± 3.0	56.1 ± 20.5	**< 0.001**
Days of hospital stay (mean ± SD)	2.7 ± 1.1	7.5 ± 3.7	**< 0.001**
Longest diameter of tumor (mm, mean ± SD)	19.5 ± 13.9	26.7 ± 13.3	0.192
Number of patients with associated diseases, N (%)	9 (81.8)	15 (100)	0.169
Previous treatment, N (%)			
Surgery	6 (54.5)	9 (60.0)	0.548
Surgery + radiotherapy	5 (45.5)	6 (40.0)	
Number of patients with lichen sclerosus, N (%)	5 (45.5)	11 (73.3)	0.228
Gradus, N (%)			
1	1 (9.1)	5 (33.3)	0.305
2	8 (72.7)	7 (46.7)	
3	2 (18.2)	3 (20.0)	
Anatomical site of tumor, N (%)			
Clitoris	1 (9.1)	2 (13.3)	0.506
Paraurethral	3 (27.3)	3 (20.0)	
Labia minora	3 (27.3)	4 (26.7)	
Labia majora	2 (18.2)	5 (33.3)	
Perineum	2 (18.2)	1 (8.3)	
Anesthesia, N (%)			
Local	4 (36.4)	8 (53.3)	0.453
General	7 (63.6)	7 (46.7)	
p16 status, N (%)			
Positive	3 (27.3)	3 (20.0)	0.509
Negative	8 (72.7)	12 (80.0)	

1 BMI = body mass index; ECT = electrochemotherapy; N = number; SD = standard deviation

No significant differences in EQ-5D scores were found between quality of life before electrochemotherapy and at each follow-up visit (Table 2).

**TABLE 2.** Mean values before and 1, 3 and 6 months after electrochemotherapy using the EuroQol - 5 Dimension (EQ-5D) questionnaire and comparison of the mean values

	Electrochemotherapy group							
	Mean (SD)				P			
	**Before ECT**	**1 month after ECT**	**3 months after ECT**	**6 months after ECT**	Before - 6 months	Before - 1 month	1 month - 3 months	3 months - 6 months
**EQ 5-D**	0.75 ± 0.21	0.62 ± 0.23	0.82 ± 0.23	0.78 ± 0.20	0.637	0.126	0.079	0.652

1 ECT = electrochemotherapy; SD = standard deviation

Table 3 summarizes the functional and symptom scales and global health status from the EORTC QLQ-C30 questionnaire for the electrochemotherapy group and the surgery group. Most patients in both groups had no problems with nausea, vomiting, constipation, diarrhea and financial difficulties, and these data were not analyzed further. In the electrochemotherapy group, there were significant improvement in the functional scales (with the exception of physical functioning), symptom scales (with the exception of dyspnea) and global health status after electrochemotherapy. A significant improvement was observed 3 months after electrochemotherapy. There was no significant difference between 3 and 6 months after electrochemotherapy. There was no significant difference in the functional and symptom scales and global health status before and after the surgical procedure.

**TABLE 3.** Functional and symptom scales and global health status data from the European Organization for Research and Treatment of Cancer Quality of Life Questionnaire Core 30 (EORTC QLQ-C30) for the electrochemotherapy and surgery groups

	Electrochemotherapy group						Surgery group					
	Before	3 months after	6 months after	Before *vs*. 6	Before *vs*. 3	3 *vs*. 6	Before	3 months after	6 months after	Before *vs*. 6	Before *vs*. 3	3 *vs*. 6
	Mean (SD)			p			Mean (SD)			p		
Functional scales											
Physical functioning	60.60 ± 14.13	75.16 ± 19.34	77.57 ± 21.14	0.148	0.116	0.748	52,89 ± 13.68	56,89 ± 15.91	58,22 ± 20.39	0.894	0.744	0.935
Role functioning	60.61 ± 13.49	78.79 ± 18.39	83.33 ± 22.36	**0.017**	0.056	0.519	57.78 ± 15.26	63.33 ± 15.69	57.78 ± 19.79	0.681	0.653	0.436
Emotional functioning	75.00 ± 8.33	95.46 ± 10.11	93.94 ± 11.84	**<0.001**	**< 0.001**	0.949	83.89 ± 10.67	90.00 ± 10.54	87.22 ±14.39 0.279	0.116	0.806
Cognitive functioning	63,64 ± 14.56	77,27 ± 13.48	81,82 ± 13.85	**0.011**	**0.034**	0,478	70,00 ± 12.91	68.89 ± 19.79	61.11 ± 25.71	0.390	0.775	0.345
Social functioning	60.61 ± 11.24	81.81 ± 11.68	84.85 ± 13.85	**< 0.001**	**< 0.001**	0.606	60.00 ± 12.28	71.11 ± 13.31	65.56 ± 17.21	0.094	0.037	0.436
**Symptom scales**											
Dyspnea	21.21 ± 12.47	9.09 ± 5.57	6.06 ± 3.48	0.09	0.133	1.000	15.55 ± 5.36	17.78 ± 7.21	17.78 ± 7.21	0.775	0.775	1.000
Pain	45.45 ± 19.85	15.15 ± 5.73	22.73 ± 10.07	**0.005**	**0.002**	0.797	46.67 ± 16.91	28.89 ± 7.21	38.89 ± 12.42	0.064	**0.023**	0.233
Fatigue	39.42 ± 16.72	20.18 ± 5.58	15.12 ± 5.14	**0.014**	**0.047**	0.519	36.31 ± 11.46	34.82 ± 13.90	41.49 ± 16.53	0.421	0.935	0.285
Insomnia	39.39 ± 13.49	9.09 ± 5.57	6.06 ± 3.48	**< 0.001**	**< 0.001**	0.748	33.33 ± 17.82	33.33 ± 17.81	35.55 ± 19.79	0.926	1.000	0.806
Appetite loss	18.18 ± 7.41	6.06 ± 3.05	6.06 ± 3.05	**< 0.001**	**< 0.001**	1.000	15.55 ± 7.21	13.33 ± 6.90	20.00 ± 6.90	0.544	0.775	0.367
**Globa health status**	49.24 ± 10.17	71.97 ± 15.49	71.21 ± 18.01	**0.002**	**< 0.001**	1.000	47.78 ± 11.56	51.11 ± 13.31	46.67 ± 18.85	0.695	0.539	0.461

1 SD = standard deviation

According to the analyses of the EORTC QLQ-C30 questionnaire, the comparison between the electrochemotherapy group and the surgery group showed a significant difference in emotional functioning before the procedure in favor of surgery. Three and six months after the procedure, there was a significant difference in physical functioning, fatigue, insomnia and global health status in favor of the electrochemotherapy group. In addition, six months after the procedure, there was a significant difference in role functioning, cognitive functioning, social functioning and appetite loss in favor of the electrochemotherapy group. A detailed comparison of the groups is shown in Table 4.

**TABLE 4.** Comparison of the mean values of the functional and symptom scales and global health status between the electrochemotherapy and surgery groups before and 3 and 6 months after the procedure using the EORTC QLQ-C30

	Before			3 months after			6 months after		
	ECT group	Surgery group	p	ECT group	Surgery group	p	ECT group	Surgery group	p
	Mean (SD)			Mean (SD)			Mean (SD		
Functional scales								
Physical functioning	60.60 ± 14.13	52,89 ± 13.68	0.164	75.16 ± 19.34	56,89 ± 15.91	**0.020**	77.57 ± 21.14	58,22 ± 20.39	**0.027**
Role functioning	60.61 ± 13.49	57.78 ± 15.26	0.721	78.79 ± 18.39	63.33 ± 15.69	0.054	83.33 ± 22.36	57.78 ± 19.79	**0.008**
Emotional functioning	75.00 ± 8.33	83.89 ± 10.67	**0.041**	95.46 ± 10.11	90.00 ± 10.54	0.097	93.94 ± 11.84	87.22 ± 14.39	0.180
Cognitive functioning	63,64 ± 14.56	70,00 ± 12.91	0.217	77,27 ± 13.48	68.89 ± 19.79	0.180	81,82 ± 13.85	61.11 ± 25.71	**0.036**
Social functioning	60.61 ± 11.24	60.00 ± 12.28	0.646	81.81 ± 11.68	71.11 ± 13.31	0.087	84.85 ± 13.85	65.56 ± 17.21	**0.011**
Symptom scales								
Dyspnea	21.21 ± 12.47	15.55 ± 5.36	0.610	9.09 ± 5.57	17.78 ± 7.21	0.258	6.06 ± 3.48	17.78 ± 7.21	0,134
Pain	45.45 ± 19.85	46.67 ± 16.91	0.799	15.15 ± 5.73	28.89 ± 7.21	0.061	22.73 ± 10.07	38.89 ± 12.42	0.054
Fatigue	39.42 ± 16.72	36.31 ± 11.46	0.878	20.18 ± 5.58	34.82 ± 13.90	**0.047**	15.12 ± 5.14	41.49 ± 16.53	**0.001**
Insomnia	39.39 ± 13.49	33.33 ± 17.82	0.507	9.09 ± 5.57	33.33 ± 17.81	**0.005**	6.06 ± 3.48	35.55 ± 19.79	0.001
Appetite loss	18.18 ± 7.41	15.55 ± 7.21	0.760	6.06 ± 3.05	13.33 ± 6.90	0.087	6.06 ± 3.05	20.00 ± 6.90	**0.009**
Globa health status	49.24 ± 10.17	47.78 ± 11.56	0.683	71.97 ± 15.49	51.11 ± 13.31	**0.009**	71.21 ± 18.01	46.67 ± 18.85	0.004

1 ECT = electrochemotherapy; SD = standard deviation

According to the EORTC QLQ-VU34 questionnaire, patients treated with electrochemotherapy had no changes in defecation or problems with leakage of stool or felt the urge to hurry to get to the toilet. In addition, there was no change or deterioration in feelings and physical attractiveness in patients treated with electrochemotherapy. There was an improvement in urinary symptoms: less pain or burning when urinating, less urine leakage and less urge to urinate. The significant difference in improvement in urination was between 1 month and 3 months after electrochemotherapy. There was also an improvement in symptoms related to scarring and mutilation of the external genitalia, with less difficulty sitting and less narrowing or tightness of the vaginal entrance. The significant difference in improvement in scarring and mutilation of the external genitalia was between 1 month and 3 months after electrochemotherapy (Table 5).

**TABLE 5.** Comparison of mean values and p values for scarring and mutilation of the external genitalia, physical attractiveness, urination and defecation symptoms before and 1, 3 and 6 months after electrochemotherapy using the EORTC QLQ-VU34

	Electrochemotherapy group							
	Mean (SD)				p			
	Before ECT	1 month after ECT	3 months after ECT	6 months after ECT	Before – 6 months	Before - 1 month	1 month – 3 months	3 months – 6 months
Scaring	6.91 ± 1.58	6.45 ± 1.92)	3.27 ± 0.90	3.27 ± 0.90	**<0.001**	0.797	**<0.001**	1.00
Physical attractiveness	3.00 ±0.00	3.00 ± 0.00	3.00 ± 0.00	3.00 ± 0.00	/	/	/	/
Urination	7.27 ± 2.05	6.09 ± 1.81	4.63 ± 1.50	4.45 ± 1.21	**0.002**	0.171	**0.04**	0.748
Defecation	2.00 ± 0.00	2.00 ± 0.00	2.00 ± 0.00	2.00 ± 0.00	/	/	/	/

1 ECT = electrochemotherapy; SD = standard deviation

**FIGURE 1.**
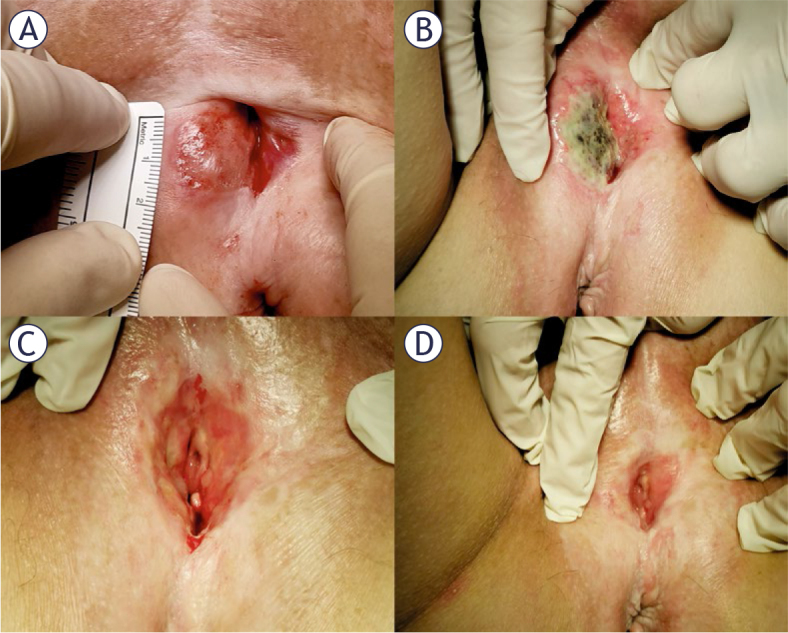
Vulvar cancer recurrence at the vaginal entrance near the urethra **(A)**. Presence of necrosis one month after treatment with electrochemotherapy **(B)**. The wound completely healed three months after treatment with electrochemotherapy **(C)**. The woman had no problems with urination, defecation and sitting six months after treatment with electrochemotherapy **(D)**.

The case of vulvar cancer recurrence at the vaginal entrance near the urethra is shown in Figure 1A. One month after electrochemotherapy, the necrosis can be seen in Figure 1B. Three months after treatment with electrochemotherapy, the wound was completely healed (Figure 1C). Six months after treatment with electrochemotherapy, the woman had no problems with urination, defecation and sitting and there were no signs of vulvar cancer (Figure 1D).

The VAS score did not differ significantly between the electrochemotherapy group and the surgical group before and 3 months and 6 months after the procedure (Table 6). Before electrochemotherapy, the mean VAS score was 2.36 ± 1.12, and before surgery, the mean VAS score was 2.21 ± 1.48. One month after electrochemotherapy, the mean VAS score was 3.64 ± 2.19. Three months after electrochemotherapy, the mean VAS score was 1.64 ± 1.57, and three months after surgery, the mean VAS score was 2.07 ± 1.07. Six months after electrochemotherapy, the mean VAS score was 1.82 ± 1.83, and six months after surgery, the mean VAS score was 2.14 ± 1.23.

**TABLE 6.** Comparison of the mean values with standard deviations and p values of the VAS score between the electrochemotherapy and surgery groups before and 3 and 6 months after the procedure

	Mean (SD)		p
VAS	ECT	Surgery	ECT *vs*. Surgery
**Before**	2.36 ± 1.12	2.21 ± 1.48	0.776
**1 month after procedure**	3.64 ± 2.19	/	/
**3 months after procedure**	1.64 ± 1.57	2.07 ± 1.07	0.442
**6 months after procedure**	1.82 ± 1.83	2.14 ± 1.23	0.620

1 ECT = electrochemotherapy; SD = standard deviation

## Discussion

To our knowledge, this is the first study to report the quality of life of women with recurrent vulvar cancer treated with electrochemotherapy for nonpalliative purposes. This study has shown that the quality of life after treatment with electrochemotherapy is comparable and in some segments better than after surgical treatment.

In the electrochemotherapy group, the EQ-5D questionnaires and the EORTC QLQ-VU34 questionnaires were associated with the lowest quality of life one month after the procedure. The main reason for this is that in most of our patients, necrosis developed at the site of the tumor two weeks after electrochemotherapy. In the case of necrosis after electrochemotherapy, tissue healing occurs secondarily over 4–10 weeks, depending on the tumor size and tumor response.^47^ In our study, the main symptom one month after electrochemotherapy, apart from necrosis, was pain, with a mean VAS score of 3.64 ± 2.19. When treating ulcerated neoplastic wounds, it is advisable to involve a specialized care team, together with a cancer pain service.^18^ In our cases, pain was treated with nonopioid and opioid peroral analgesics, and cancer pain services were not needed. In general, the treated skin can be covered with nonadherent, comfortable dressings during the inflammatory phase, whereas ulcerated lesions are best treated with advanced dressings such as alginates, charcoal and silver. Tissue necrosis can be treated by enzymatic and/or surgical debridement to prevent superinfection and promote healing, according to standard operating procedures for electrochemo-therapy.^45^ In all patients in the electrochemotherapy group, the wounds were completely healed three months after treatment. Three months after electrochemotherapy, the mean VAS score was 1.64 ± 1.57, which was lower than that in the surgical group, where it was 2.07 ± 1.07.

In patients treated with electrochemotherapy, the EORTC QLQ-VU34 questionnaire showed that electrochemotherapy did not cause problems with defecation, even when the tumor was located in close proximity to the anal sphincter and anal canal. This is the unique advantage of electrochemotherapy because of its selectivity for cancer cells while sparing the surrounding healthy tissue.^29^ Surgical removal of the tumor, which is located in the immediate vicinity of the anal sphincter muscle and the anal canal, can lead to impaired defecation with leakage of stool and, in the worst case, to the formation of an intestinal stoma.^48^ The EORTC QLQ-VU34 questionnaire showed that electrochemotherapy did not worsen feelings and physical attractiveness. All women in the electrochemotherapy group were not sexually active in the six months following the procedure. According to the literature, vulvar cancer has a role in worsening sexual functioning with a disruption and reduction in sexual activity, a lower quality of partner relationships and maritial satisfaction. Sexual dysfunction is closely related to anxiety and depressive symptoms.^12^ Owing to the consequences of surgery, psychological stress may be related to altered body image. Women may experience shame and embarrassment associated with severe social stigmatization, which can lead to feelings of isolation and loss of self-esteem.^12^ The EORTC QLQ-VU34 questionnaire showed that women treated with electrochemotherapy had improved urinary symptoms. Six months after electrochemotherapy treatment, none of the patients showed signs of urinary incontinence. The EORTC QLQ-VU34 questionnaire also showed an improvement in symptoms related to scarring and mutilation of the external genitalia after electrochemotherapy. Since the surrounding healthy tissue is spared during electrochemotherapy and no sutures are used as in surgery, the mutilation of the external genitalia is less pronounced after electrochemotherapy, and the narrowing and scarring of the vaginal entrance is also less pronounced. Significant improvement was observed between one and three months after electrochemotherapy, which was due to the presence of necrosis one month after electrochemotherapy. Three months after electrochemotherapy, the women no longer had difficulty sitting, which is very important for daily life.

The EORTC QLQ-C30 questionnaire showed a significant improvement in quality of life three months after electrochemotherapy. There was no significant improvement in quality of life between three and six months after electrochemotherapy. The EQ-5D and EORTC QLQ-VU34 questionnaires also showed no significant improvement in quality of life between three and six months after electrochemotherapy. These results indicate that an improvement in quality of life can be expected three months after electrochemotherapy and that no improvement or deterioration in quality of life can be expected later.

The comparison of the EORTC QLQ-C30 questionnaires between the electrochemotherapy group and the surgery group showed that three months after the procedure, the women treated with electrochemotherapy were able to take a longer walk, needed to stay in bed or in a chair less during the day and required less help with eating, dressing and washing. These women also slept better and were less tired. In addition, six months after the procedure, the women treated with electrochemotherapy were able to perform normal daily activities and hobbies, had less difficulty concentrating on things, had better family life and a better appetite. There is a lack of scientific evidence on the quality of life of patients with vulvar cancer. It is therefore very difficult to compare our results with those of electrochemotherapy treatment with those that can be achieved with other therapies in this situation.^44,49^

The main limitation of the study is the small number of patients included. However, vulvar cancer recurrences are rare and difficult to treat. Therefore, even small prospective studies are important to verify the value of new treatment methods. Another limitation of the study is the short-term follow-up, but our study suggests an improvement in quality of life three months after electrochemotherapy and that no improvement or worsening of quality of life is expected later. Our study showed that six months after treatment, the quality of life of patients treated with electrochemotherapy was no worse than the quality of life of patients treated with surgery. Despite some limitations of this study, this is the first study to provide information on the quality of life of women treated with electrochemotherapy for nonpalliative purposes in vulvar cancer recurrence. Furthermore, the patients in the electrochemotherapy group and the surgical group were homogeneous and had similar clinical characteristics, histologic types, previous treatments, and anatomic sites.

In conclusion, our study showed that the quality of life after treatment with electrochemotherapy is comparable and in some segments even better than after surgical treatment. Electrochemotherapy is suggested in cases where the recurrence of vulvar cancer occurs in close proximity to the urethra, anus or vaginal entrance to improve quality of life and avoid mutilating surgery on the external genitalia. However, further studies are needed to analyze and identify the best candidates for this promising treatment. Owing to the rarity of vulvar cancer, centralization of the treatment of this disease is necessary.
